# Apoptotic-mimetic nanovesicles orchestrate immune–vascular–osteogenic crosstalk for critical-sized craniofacial bone regeneration

**DOI:** 10.1016/j.mtbio.2026.103119

**Published:** 2026-04-13

**Authors:** Hao Pan, Min Zhang, Likai Chen, Haoze Zhu, Siman Huang, Yueyue Huang, Yiyu Li, Zuchang Liu, Xiaokun Li, Cailong Liu

**Affiliations:** a Department of Orthopaedic Surgery, Department of Wound Healing, The First Affiliated Hospital of Wenzhou Medical University, Wenzhou, Zhejiang, 325000, China; b National Key Laboratory of Macromolecular Drugs and Large-scale Preparation, School of Pharmaceutical Sciences, Wenzhou Medical University, Wenzhou, Zhejiang, 325035, China; c Key Laboratory of Intelligent Treatment and Life Support for Critical Diseases of Zhejiang Province, Department of Intensive Care Unit, The First Affiliated Hospital of Wenzhou Medical University, Wenzhou, Zhejiang, 325000, China; d Department of Clinical Biochemistry and Molecular Diagnostics, College of Medical Technology, Tianjin Medical University, Tianjin, 300203, China; e Shanghai Engineering Research Center of Tooth Restoration and Regeneration & Tongji Research Institute of Stomatology & Department of Dental Implantation, Shanghai Tongji Stomatological Hospital and Dental School, Tongji University, Shanghai, 200072, China

**Keywords:** Apoptotic nanovesicles, Bone regeneration, Immunomodulation, Osteogenesis, Angiogenesis

## Abstract

The management of critical-sized craniomaxillofacial bone defects remains a formidable clinical challenge due to the concomitant issues of prolonged inflammation, inadequate vascularization, and insufficient endogenous osteogenesis. Herein, a novel cell-free bone regeneration strategy is reported based on biomimetic apoptotic nanovesicles (Apo-NVs) derived from apoptotic T-lymphocytes membrane. The Apo-NVs, which mimic the "find-me" and "eat-me" signals of natural apoptotic cells, are engineered into a gelatin methacryloyl (GelMA) hydrogel for sustained localized delivery. In vitro, the Apo-NVs demonstrate superior immunomodulatory efficacy by promoting the repolarization of pro-inflammatory M1 macrophages towards a pro-healing M2 phenotype. Concurrently, they directly enhance the osteogenic differentiation of bone marrow-derived mesenchymal stem cells (BMSCs) and potently stimulate human umbilical vein endothelial cells (HUVECs) proliferation, migration, and tube formation. In vivo, the GelMA-Apo-NVs significantly augments bone regeneration and vascularization compared to GelMA and GelMA-NVs groups, as validated by micro-computed tomography and histological analyses. Mechanistic unraveling through transcriptomic profiling reveals that the regenerative function of Apo-NVs is orchestrated through the activation of Wnt/β-catenin signaling pathway. This activation not only directly drives osteogenic gene expression but also upregulates *vegfa*, thereby coupling angiogenesis with osteogenesis, while simultaneously inhibiting the NF-κB pathway to inhibit inflammation. This study pioneers a versatile apoptotic vesicle-based platform that harmonizes the immune-osteogenic-vascular triad, presenting a potent and promising therapeutic paradigm for regenerative medicine.

## Graphical abstract

**Figure:**
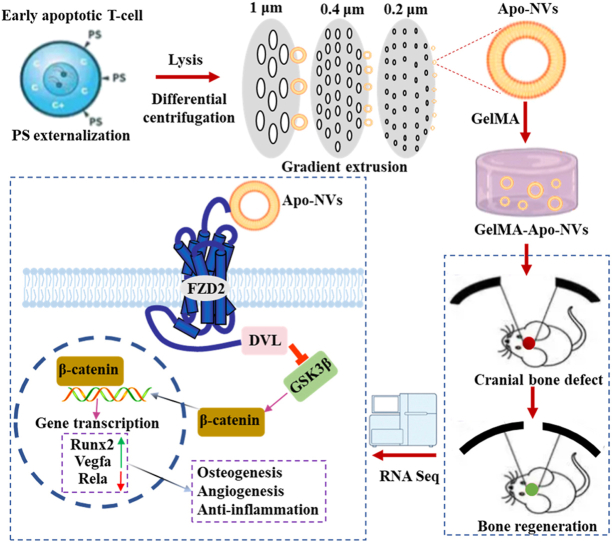

## 1 Introduction

The regeneration of critical-sized craniomaxillofacial bone defects resulting from trauma, oncological resection, or congenital anomalies remains a formidable challenge in clinical practice [1,2]. The inherent limitations of autografts (e.g., donor site morbidity, limited availability) and allografts (e.g., immune rejection, risk of disease transmission) have spurred intensive research into the development of advanced biomaterial-based strategies and tissue engineering approaches [3,4]. While biomaterial scaffolds provide essential structural support, the complex regenerative process demands a meticulously orchestrated biological response including anti-inflammation and effective osteogenesis and vascularization [[5], [6], [7]]. A persistent pro-inflammatory macrophage phenotype disrupts this harmony, leading to a hostile microenvironment that impedes vascular ingrowth and suppresses the osteogenic differentiation of mesenchymal stem cells (MSCs), ultimately resulting in fibrotic non-union [8,9]. Consequently, research is increasingly pivoting to the design of immunomodulatory biomaterials that can actively manipulate the local immune milieu to foster a pro-regenerative state, rather than merely providing structural support.

Recent breakthroughs in engineered extracellular vesicle have unveiled their profound potential as bone regeneration therapeutics, capable of transporting bioactive molecules to direct cell fate and tissue repair [[10], [11], [12]]. Particularly, vesicles derived from apoptotic cells have garnered significant interest, as they naturally inherit "find-me" and "eat-me" signals, such as phosphatidylserine (PS) exposure, which play a crucial role in promoting efferocytosis and initiating inflammation resolution [13,14]. Apoptotic vesicles are increasingly recognized not as mere cellular debris but as key orchestrators of tissue homeostasis, capable of driving macrophage polarization toward an anti-inflammatory, pro-repair M2 phenotype [[15], [16], [17]]. For instance, apoptotic vesicles derived from human bone marrow mesenchymal stem cells have been demonstrated to enhance osteogenesis, suppress osteoclast formation, ameliorate osteoporosis, and stimulate bone regeneration in defect areas [18]. Furthermore, apoptotic extracellular vesicles loaded onto phospholipid-modified titanium surfaces were shown to promote early angiogenesis and enhance implant osseointegration through effective immune regulation [19]. However, their clinical translation faces considerable challenges due to inherent heterogeneity, limited scalable production, and poor stability. These limitations have prompted the development of engineered biomimetic alternatives. Recent advances in nano-biotechnology have demonstrated that vesicles derived from cell membranes—particularly from immunologically active sources—can effectively replicate the surface biology of their parent cells while offering superior compositional control, scalability, and stability [[20], [21], [22]].

Herein, we presented a novel cell-free bone regeneration strategy based on biomimetic apoptotic nanovesicles (Apo-NVs) derived from apoptotic T-lymphocytes membrane. The immunomodulatory, angiogenic, and osteogenic efficacy of Apo-NVs was systematically evaluated through a combination of in vitro studies and a murine critical-sized calvarial defect model. Furthermore, transcriptomic profiling was conducted to reveal the underlying molecular mechanisms governing the regenerative effects. This study aims to pioneer a versatile apoptotic vesicle-based platform that harmonizes the immune-osteogenic-vascular triad, offering a promising therapeutic paradigm for advanced bone tissue engineering.

## 2 Methods and materials

### 2.1 Preparation of Apo-NVs

Jurkat cells (human T lymphocyte cell line) were acquired from the Leibniz Institute DSMZ (Germany) and cultured in RPMI 1640 medium (Gibco, USA), supplemented with 10% fetal bovine serum (FBS; Biowest, France) and 1% penicillin/streptomycin solution (Sigma-Aldrich, USA). Cells were maintained at 37 °C in a humidified 5% CO_2_ incubator. To induce apoptosis, cells were treated with 0.5 μM staurosporine (STS; Abcam, UK) for 12 h. Following induction, cells were harvested by centrifugation at 200×*g* for 10 min at 4 °C and washed twice with ice-cold phosphate-buffered saline (PBS), and then resuspended in a hypotonic Tris-Magnesium (TM) buffer (10 mM Tris-HCl, pH 7.4, 10 mM MgCl_2_) containing 1 × protease inhibitor cocktail (Roche, Switzerland) and incubated overnight at 4 °C under gentle agitation. The cells were then homogenized using a pre-cooled tight-fitting glass Dounce homogenizer (DWK Life Sciences, USA) with 20 complete strokes. The homogenate was subjected to two successive centrifugation steps at 3000×*g* for 10 min at 4 °C to remove nuclei and intact cells. The resulting supernatant was ultracentrifuged at 100,000×*g* for 45 min at 4 °C using a Type 70 Ti rotor (Beckman Coulter, USA) to isolate plasma membrane fragments. The membrane was washed once in TM buffer, reconstituted in PBS, and stored at −80 °C. The protein concentration of the membrane preparation was quantified using a bicinchoninic acid (BCA) protein assay kit (Merck, Germany). For the generation of Apo-NVs, the membrane suspension was extruded through a series of polycarbonate membranes with pore sizes of 1000, 400, and 200 nm (Nuclepore, Whatman, UK) using a mini-extruder apparatus (Avanti Polar Lipids, USA). The extrusion process was repeated more than 15 times for each membrane under sterile conditions.

### 2.2 Characterization of Apo-NVs

The morphological features of Apo-NVs were examined via transmission electron microscopy (TEM; Thermo Fisher Scientific, USA). Particle size distribution and zeta potential were measured by dynamic light scattering (DLS) using a Zetasizer Nano ZSP instrument (Malvern Panalytical, UK). Apoptotic markers were assessed using an Annexin V–FITC/propidium iodide (PI) apoptosis detection kit (Sigma-Aldrich, USA). Fluorescence imaging was performed with a confocal laser scanning microscope (CLSM; TCS SP8, Leica Microsystems, Germany), and quantitative apoptosis analysis was carried out via flow cytometry (BD Biosciences, USA).

### 2.3 Cellular uptake of Apo-NVs

Macrophages (RAW264.7), BMSCs, and human umbilical vein endothelial cells (HUVECs) were cultured in confocal dishes. Apo-NVs were pre-labeled with DID fluorescent dye (Thermo Fisher Scientific, USA) and cultured with cells for 4 h. Cells were then washed with PBS, fixed in 4% paraformaldehyde (Electron Microscopy Sciences, USA), and stained with DAPI (Sigma-Aldrich, USA) and phalloidin-FITC (Cytoskeleton, USA) for nucleus and F-actin visualization, respectively. Images were acquired using a confocal laser scanning microscope.

### 2.4 Cell Counting Kit-8 assay

Cell viability was assessed using a Cell Counting Kit-8 (CCK-8; Dojindo Molecular Technologies, Japan). Cells were seeded into 96-well plates at a density of 3 × 10^3^ cells per well and treated with NVs or Apo-NVs (20 μg/mL) for 72 h. Then, 10 μL of CCK-8 reagent was introduced into each well and incubated for 4 h at 37 °C. Absorbance was recorded at 450 nm using a multimode microplate reader (SpectraMax iD5, Molecular Devices, USA).

The working concentration of vesicles (20 μg/mL, protein equivalent) was selected as a commonly used mid-range dose in EV/nanovesicle functional assays for macrophage polarization, osteogenic differentiation, and endothelial angiogenesis readouts, facilitating comparison with prior EV-hydrogel bone-regeneration studies using similar endpoints [23,24].

### 2.5 In vitro macrophage polarization

RAW264.7 cells were cultured in RPMI 1640 medium with 10% FBS and 1% penicillin/streptomycin. To induce a pro-inflammatory phenotype, cells were stimulated with lipopolysaccharide (LPS; 500 ng/mL; InvivoGen, USA) for 24 h. Subsequently, cells were treated with NVs or Apo-NVs (20 μg/mL) for 48 h. Polarization was assessed by immunofluorescence for protein levels of M1 (TNF-α) and M2 (IL-10) markers, and by qPCR for mRNA levels of M1 (*Tnf-α, Il-1β*) and M2 (*Tgf-β, Il-10*) markers.

### 2.6 RNA extraction and quantitative real-time PCR

Total RNA was extracted using the RNeasy Mini Kit (Qiagen, Germany) and quantified with a NanoDrop One spectrophotometer (Thermo Fisher Scientific, USA). Complementary DNA (cDNA) was synthesized using the High-Capacity cDNA Reverse Transcription Kit (Applied Biosystems, USA). qPCR was performed on a QuantStudio 6 Pro system (Applied Biosystems, USA) using PowerUp SYBR Green Master Mix (Applied Biosystems, USA). Primer sequences are listed in Tables S1–3.

### 2.7 Immunofluorescence staining

Samples were fixed in 4% paraformaldehyde, permeabilized with 0.4% Triton X-100, and blocked with 5% normal donkey serum. Primary antibodies against BMP2 (Abcam, UK), RUNX2 (Abcam, UK), CD31 (Abcam, UK), TNF-α (Cell Signaling Technology, USA), and IL-10 (R&D Systems, USA) were applied overnight at 4 °C. After washing, samples were incubated with fluorophore-conjugated secondary antibodies (Jackson ImmunoResearch, USA) and counterstained with DAPI.

### 2.8 Culture of bone marrow-derived mesenchymal stem cells

Murine bone marrow-derived mesenchymal stem cells (BMSCs) were obtained from Cyagen Biosciences (USA) and authenticated by flow cytometric analysis of characteristic surface markers. Cells were cultured in MEM Alpha medium (Cytiva, USA) containing 10% FBS and 1% penicillin/streptomycin at 37 °C in a 5% CO_2_ atmosphere. Culture medium was refreshed every 24 h, and cells were passaged at 80–90% confluence every third day. For osteogenic induction, BMSCs were cultured in osteogenic medium composed of MEM Alpha, 10% FBS, 1% penicillin/streptomycin, 50 μM ascorbic acid (Sigma-Aldrich, USA), 20 mM β-glycerophosphate (STEMCELL Technologies, Canada), and 1 μM dexamethasone (MedChemExpress, USA). The induction medium was replaced every 48 h.

### 2.9 Analysis of apoptosis

Apoptosis was quantified with an Annexin V–FITC/PI detection kit. Following 72 h of treatment with either NVs or Apo-NVs (20 μg/mL), cells were harvested by trypsinization, washed with PBS, and stained according to the manufacturer's instructions. Flow cytometric analysis was conducted on a FACSCanto II system (BD Biosciences, USA), and data were analyzed using FlowJo software.

### 2.10 Live/dead staining

Cellular viability was evaluated using a Calcein-AM/PI double-stain kit (Thermo Fisher Scientific, USA). After 72 h of treatment with NVs or Apo-NVs (20 μg/mL), cells were incubated with Calcein-AM and PI working solutions for 30 min at 37 °C. Fluorescence images were captured using an inverted fluorescence microscope (Nikon Eclipse Ti2, Japan).

### 2.11 BMSCs osteoblastic differentiation assay

To evaluate early osteogenic differentiation, BMSCs were cultured in osteogenic induction medium for 7 days. The cells were subsequently fixed with 4% paraformaldehyde for 15 min at room temperature and permeabilized with 0.1% Triton X-100 for 10 min. Alkaline phosphatase (ALP) staining was detected using an ALP kit (Sigma, Germany). For quantitative analysis, ALP enzymatic activity was measured using the Alkaline Phosphatase Activity Assay Kit (Beyotime Biotechnology, Shanghai) following the manufacturer's instructions. The absorbance was recorded at 405 nm using a multimode microplate reader.

Mineralization capacity, indicative of late-stage osteogenic differentiation, was assessed through Alizarin Red S (ARS) staining after 14 days of culture in osteogenic induction medium. The cells were fixed with 4% paraformaldehyde for 30 min at room temperature and gently washed three times with distilled water to remove residual salts. The fixed cells were then stained with 2% Alizarin Red S solution (pH 4.2; Solarbio Science & Technology Co., Ltd., China) for 20 min at room temperature. Following staining, unbound dye was removed by extensive washing with distilled water, and the stained mineralized nodules were visualized under a phase-contrast microscope. For quantitative analysis, the bound dye was solubilized with 10% cetylpyridinium chloride (CPC; Meilun Biotech, China) for 30 min at room temperature. The absorbance of the solubilized solution was measured at 562 nm using the multimode microplate reader.

### 2.12 Transwell migration assay

Cells migration was evaluated using 8 μm pore-size Transwell inserts (Corning, USA). HUVECs suspended in serum-free medium were placed in the upper chamber, while the lower chamber contained medium with 10% FBS. After 8 h, migrated cells on the lower surface were fixed and stained with 0.2% crystal violet. For quantification, membranes were imaged at a fixed magnification in five random fields per insert. Migrated cells were counted using ImageJ and the mean value per insert was used for statistics.

### 2.13 Tube formation assay

A tube formation assay was conducted using Matrigel (Corning, USA). HUVECs pretreated with NVs or Apo-NVs were seeded onto Matrigel in 96-well plates. Tubular structures were imaged after 4 h, and network parameters were analyzed with ImageJ software.

### 2.14 EdU assay

Proliferation was assessed using a Click-iT EdU Imaging Kit (Thermo Fisher Scientific, USA). HUVECs were treated with 10 μM EdU for 24 h, fixed, and stained according to the manufacturer's instructions. EdU-positive cells were visualized via confocal microscopy. EdU-positive nuclei and total nuclei (DAPI) were quantified from five random fields per well using ImageJ cell counter. The EdU incorporation rate was calculated as EdU% = (EdU^+^ nuclei/total nuclei) × 100%.

### 2.15 Preparation and characterization of GelMA–Apo-NVs

Gelatin methacryloyl (GelMA; Sigma-Aldrich, USA) was dissolved at 10% (w/v) in PBS. Apo-NVs were loaded into GelMA at 5 × 10^11^ particles per implant (corresponding to 50 μL of 1 × 10^11^ particles/mL vesicle suspension incorporated into 0.5 mL GelMA). This local loading magnitude is comparable to doses used in EV-hydrogel cranial bone-repair studies (typically tens to hundreds of micrograms EV protein per hydrogel/scaffold or similar particle levels) designed for sustained retention and bioactivity at defect sites [[23], [24], [25]]. Photo-crosslinking was initiated by UV exposure (365 nm, 5 mW/cm^2^) for 60 s. The microstructure of lyophilized hydrogels was examined using field-emission scanning electron microscopy (FE-SEM; JEOL JSM-7800F, Japan).

To avoid interference from GelMA (a protein-based polymer) in protein-assay readouts, the release profiles of Apo-NVs from GelMA were quantified using a fluorescence-labeling method [[26], [27], [28]]. Briefly, Apo-NVs were labeled with a lipophilic membrane dye (DID according to the manufacturer's protocol). Unbound dye was removed by ultracentrifugation (100,000×*g*, 45 min, 4 °C) and washing, and the labeled Apo-NVs were resuspended in PBS. Labeled Apo-NVs were then encapsulated into GelMA hydrogels as described above. Each hydrogel was incubated in PBS at 37 °C under gentle shaking. At predetermined time points, the supernatant was collected and replaced with an equal volume of fresh buffer. The fluorescence intensity of the collected supernatants was measured using a microplate reader at the corresponding excitation/emission wavelengths of the dye. A standard curve was generated using serial dilutions of the same labeled Apo-NV preparation (with known particle number and/or protein equivalent), allowing conversion of fluorescence intensity to released Apo-NV amount. Cumulative release (%) was calculated as (cumulative released Apo-NV amount/total Apo-NV amount loaded) × 100%. Blank GelMA hydrogels (without Apo-NVs) and dye-only controls processed identically were included to correct background/autofluorescence and exclude potential free-dye artifacts.

### 2.16 Animal experiments

All the animal experiments were complied with the guidelines of the Tianjin Medical Experimental Animal Care, and animal protocols were approved by the Institutional Animal Care and Use Committee of Yi Shengyuan Gene Technology (Tianjin) Co., Ltd. (protocol number YSY-DWLL-2025880). Critical-sized calvarial defects (5 mm diameter) were created in 8-week-old male C57 mice and implanted with test materials. After 12 weeks, bone regeneration was evaluated via micro-CT (Skyscan 1276, Bruker, USA), and parameters including bone volume fraction (BV/TV) and bone mineral density (BMD) were calculated.

To visualize neovascularization, mice were anesthetized and systemically perfused via the left ventricle with heparinized PBS to remove blood, followed by 4% paraformaldehyde fixation. A radiopaque silicone rubber contrast agent (MICROFIL® MV-122, Flow Tech, Inc., Carver, MA, USA) was then slowly injected until effluent from the right atrium turned opaque. Samples were stored at 4 °C to allow polymerization, decalcified in 10% EDTA (3 weeks), and scanned by micro-CT. Vessel segmentation was performed using CTAn (Bruker microCT) with a consistent global threshold, and vascular volume density (vessel volume/total volume) was calculated.

Specimens were decalcified, paraffin-embedded, and sectioned at 6 μm thickness. Sections were stained with hematoxylin and eosin (H&E), Masson's trichrome, and Van Gieson (VG).

### 2.17 RNA sequencing

For tissue-level RNA-seq, animals were euthanized at 12 weeks post-implantation, and the calvarial defect region (5-mm diameter including the regenerated tissue) was carefully dissected to minimize contamination from surrounding native bone. Samples were snap-frozen in liquid nitrogen and homogenized in lysis buffer. Total RNA was extracted using the RNeasy Mini Kit (Qiagen, Germany), and sequenced on Beijing Genomics Institute. Significantly differentially expressed genes were defined using the Benjamini–Hochberg false discovery rate (FDR <0.05) with an absolute log_2_ fold-change cutoff (|log_2_FC| ≥ 1). Bioinformatic analyses including KEGG, GSEA, and PPI network analysis were performed.

### 2.18 ELISA assay

Following the indicated treatments, conditioned media were collected and clarified by centrifugation (1000×*g*, 20 min) to remove cellular debris. TNF-α and IL-10 in the resulting cell-free supernatants were quantified using commercial ELISA kits (R&D Systems, USA) in accordance with the manufacturer's protocol. Absorbance was recorded on a microplate reader, and cytokine concentrations were interpolated from calibration curves constructed with recombinant cytokine standards.

### 2.19 Statistical analysis

Data are presented as mean ± standard deviation (SD). Statistical analyses were performed using GraphPad Prism 9.5. For comparisons among three or more groups, one-way analysis of variance (one-way ANOVA) was conducted, followed by Tukey's multiple comparisons test for post hoc pairwise comparisons. A p-value <0.05 was considered statistically significant (∗p < 0.05, ∗∗p < 0.01, ∗∗∗p < 0.001).

## 3 Results and discussion

### 3.1 Preparation and characterization of Apo-NVs

**Fig. 1:**
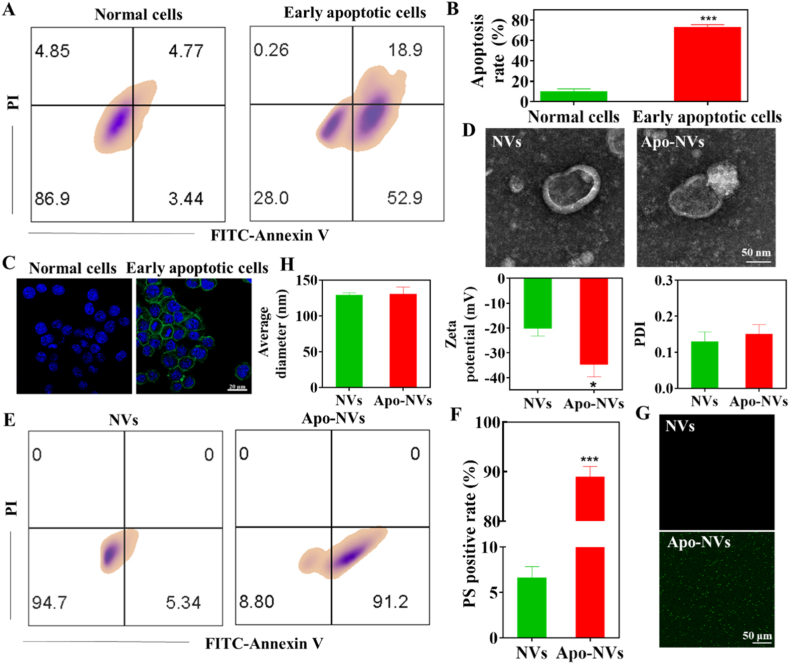
Characterization of NVs and Apo-NVs. (A) Flow cytometric quantification of apoptosis in Jurkat cells treated with 0.5 μM STS. (B) The apoptosis rate of Jurkat cells according to (A). (C) Fluorescence micrographs of apoptotic Jurkat cells stained with Annexin V-FITC and PI. (D) Representative TEM images illustrating the morphology and bilayer membrane structure of NVs and Apo-NVs. (E) Flow cytometric evaluation of phosphatidylserine exposure via Annexin V-FITC binding to Apo-NVs. (F) Percentages of PS positive NVs and Apo-NVs according to the flow cytometric analysis results of (E). (G) Confocal microscopy images showing Annexin V-FITC^+^ Apo-NVs. (H) Hydrodynamic diameter, zeta potential, and polydispersity index (PDI) of NVs and Apo-NVs. Data represent mean ± SD; ∗p < 0.05, ∗∗∗p < 0.001 versus NVs group; n = 3.

In this work, biomimetic Apo-NVs were engineered from the plasma membrane of apoptotic T lymphocytes, selected for their intrinsic immunomodulatory properties and documented homing capacity toward inflammatory sites—a trait potentially inherited by their membrane derivatives [29,30]. Apoptosis was robustly induced by treating Jurkat cells with 0.5 μM STS, a potent and well-established protein kinase inhibitor that triggers the intrinsic apoptotic pathway. The successful induction of apoptosis was quantitatively and qualitatively verified via flow cytometry and fluorescence microscopy using a dual-staining protocol with FITC-conjugated Annexin V and PI. As depicted in Fig. 1A–C, STS treatment resulted in a high proportion of apoptotic cells, predominantly in the early apoptotic stage (Annexin V^+^/PI^−^), confirming minimal secondary necrosis and ensuring membrane integrity for subsequent vesicle production.

Apo-NVs were fabricated from isolated plasma membranes of early-apoptotic Jurkat cells using a multi-step procedure involving hypotonic lysis, mechanical homogenization, differential centrifugation, and serial extrusion through polycarbonate membranes of diminishing pore sizes (1000, 400, and 200 nm). This process yielded monodisperse, nanosized vesicles. TEM imaging revealed that both control NVs and Apo-NVs exhibited the characteristic spherical or cup-shaped morphology with a well-defined bilayer structure, consistent with natural extracellular vesicles (Fig. 1D). Crucially, flow cytometric analysis demonstrated that over 90% of the generated Apo-NVs were Annexin V^+^/PI^−^ (Fig. 1E and F), attesting to the efficient preservation and surface exposure of PS—a canonical "eat-me" signal indicative of apoptotic membranes. This finding was further corroborated by confocal microscopy (Fig. 1G).

Comprehensive physicochemical characterization was performed using DLS. Both NV types displayed a narrow size distribution with a mean hydrodynamic diameter of approximately 130 nm and a low polydispersity index (PDI <0.2), confirming high sample homogeneity (Fig. 1H). Zeta potential measurements revealed strongly negative surface charges for both preparations, with Apo-NVs exhibiting a significantly more negative value (−34.7 mV) compared to NVs (−20.1 mV) (Fig. 1H). This enhanced electronegativity is a direct consequence of the increased surface exposure of anionic phospholipids, particularly phosphatidylserine, which is systematically externalized during early apoptosis. Collectively, these data affirm the successful fabrication of monodisperse, PS-presenting Apo-NVs with biophysical properties conducive to cellular interactions and downstream bioactivity.

### 3.2 Apo-NVs promote macrophage repolarization in vitro

The successful regeneration of craniomaxillofacial bone defects is critically dependent on the timely resolution of the initial inflammatory response and the establishment of a pro-regenerative microenvironment. A pivotal event in this process is the phenotypic shift of macrophages from a pro-inflammatory (M1) to an anti-inflammatory, pro-repair (M2) state. Sustained M1 polarization, characterized by the secretion of cytokines such as TNF-α and IL-1β, perpetuates inflammation, inhibits osteogenic differentiation of mesenchymal stem cells, and fosters a fibrotic environment that impedes vascularization and new bone formation. Therefore, we first investigated the capacity of Apo-NVs to modulate macrophage polarization as a potential mechanism for mitigating inflammation and promoting healing.

**Fig. 2:**
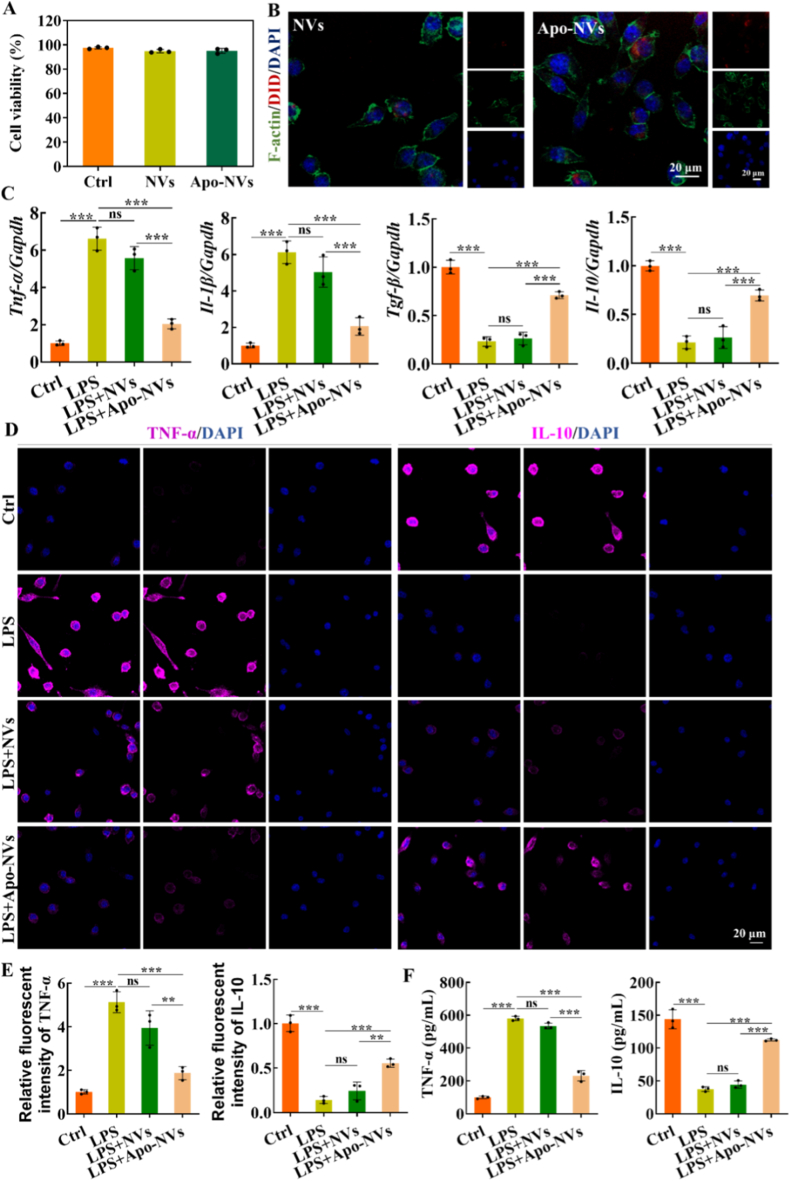
Immunomodulatory effects of Apo-NVs on macrophages. (A) Viability of RAW264.7 macrophages treated with NVs or Apo-NVs, assessed via CCK-8 assay. (B) Confocal microscopy images showing cellular internalization of DID-labeled NVs and Apo-NVs (red) by macrophages. Cytoskeleton and nuclei were stained with phalloidin (green) and DAPI (blue), respectively. (C) qPCR analysis of M1 (*Tnf-α, Il-1β*) and M2 (*Tgf-β, Il-10*) macrophage polarization markers. (D) Representative immunofluorescence images of TNF-α and IL-10 expression in macrophages. (E) The relative fluorescence intensity of TNF-α and IL10 in Fig. 2D. (F) Secretion of TNF-α and IL-10 quantified by ELISA. Data are presented as mean ± SD; ∗∗p < 0.01, ∗∗∗p < 0.001; ns, not significant.

Prior to assessing immunomodulatory function, the cytocompatibility of NVs and Apo-NVs with RAW264.7 macrophages was confirmed. Cell Counting Kit-8 assays revealed no significant cytotoxicity upon treatment with either NV type, establishing a foundation for subsequent bioactivity studies (Fig. 2A). As efficient cellular internalization is a prerequisite for the intracellular delivery of immunomodulatory cues, we next examined the uptake of fluorescently labeled vesicles. Confocal microscopy and flow cytometry analysis demonstrated significantly enhanced internalization of Apo-NVs by macrophages compared to their non-apoptotic counterparts (NVs) (Fig. 2B–Fig. S1). This preferential uptake is likely mediated by the prominent exposure of phosphatidylserine on the Apo-NVs surface, which serves as a canonical "eat-me" signal for phagocytic cells such as macrophages, facilitating efferocytosis and the subsequent initiation of anti-inflammatory signaling pathways.

To quantitatively evaluate the immunomodulatory potency of Apo-NVs, we employed an in vitro model of inflammation by pre-stimulating RAW264.7 macrophages with LPS to induce a classical M1 phenotype. Subsequent treatment with Apo-NVs effectively reversed the M1 transcriptional signature and induced a pronounced M2-associated gene expression profile. qPCR analysis demonstrated a marked downregulation of M1 genes (*Tnf-α* and *Il-1β*) and a concurrent upregulation of key M2 genes (*Tgf-β* and *Il-10*) in the Apo-NVs-treated group, an effect that was significantly more pronounced than that induced by NVs (Fig. 2C). This shift in gene expression was corroborated at the protein level by immunofluorescence staining, which visualized a clear attenuation of TNF-α signal and a robust enhancement of IL-10 expression (Fig. 2D and E). ELISA assays revealed that TNF-α levels was significantly decreased, whereas IL-10 secretion was increased in the Apo-NVs group relative to NVs. Collectively, these findings substantiate that Apo-NVs potently drive macrophage repolarization from a pro-inflammatory M1 toward a pro-healing M2 phenotype, fostering a conducive environment for the subsequent stages of bone regeneration, including osteogenesis and angiogenesis.

Although the pro-resolving immunomodulatory effect of apoptotic vesicles is not exclusive to a single parental cell type, we selected apoptotic T-lymphocyte membranes to engineer Apo-NVs for two reasons. First, exposed phosphatidylserine on apoptotic membranes is a broadly conserved “eat-me” signal that facilitates efferocytosis and inflammation resolution, thereby favoring M2-like polarization and attenuated NF-κB activity in macrophages [[31], [32], [33]]. Hence, Apo-vesicles derived from multiple cell types may share a PS-driven immunomodulatory axis, consistent with the notion that PS represents a global immunosuppressive/pro-resolving cue [33]. Second, T lymphocytes express adhesion molecules (e.g., LFA-1) that critically regulate leukocyte adhesion and trans-endothelial migration during immune surveillance and inflammation [34,35]. We reasoned that a T-cell membrane origin may enhance interactions with macrophages/endothelium in inflammatory microenvironments, potentially benefiting local retention and immune modulation after implantation. In addition, Jurkat cells provide a scalable and reproducible source for membrane production under defined culture conditions. Importantly, we acknowledge that parental cell source can influence membrane protein repertoires and associated cargos, which may affect targeting and potency. Systematic comparisons of Apo-NVs derived from different cell types (e.g., MSCs, endothelial cells, or other immune cells) represent an important direction for future work to optimize efficacy and specificity.

### 3.3 Apo-NVs enhance osteogenic differentiation of BMSCs

The direct engagement of mesenchymal stem cells and the potentiation of their osteogenic lineage commitment are pivotal for effective bone regeneration. We therefore sought to investigate the direct osteoinductive effects of Apo-NVs on BMSCs, which serve as the primary cellular source for new bone formation.

**Fig. 3:**
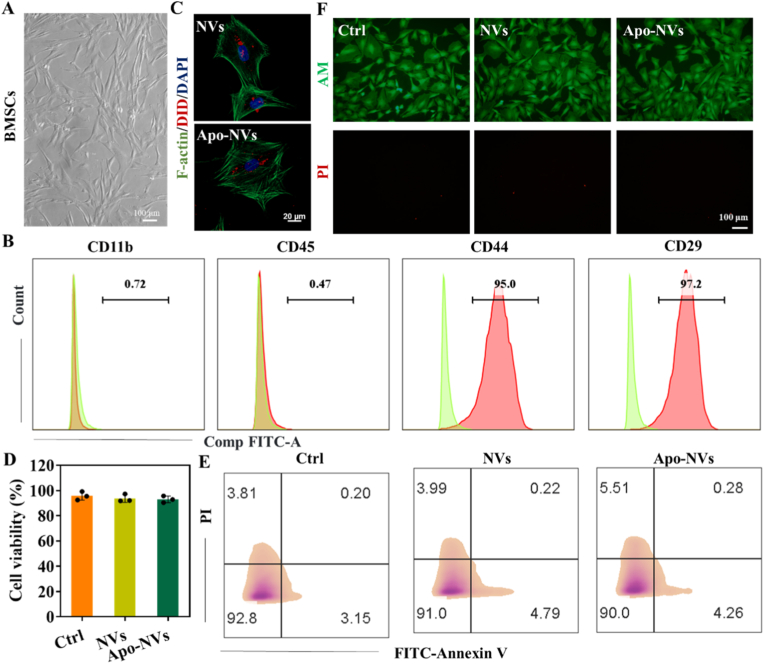
Biocompatibility of NVs and Apo-NVs with BMSCs. (A) Morphology of BMSCs observed under phase-contrast microscopy. (B) Flow cytometric analysis of BMSC surface markers (CD11b, CD45, CD44, CD29). (C) In vitro cellular uptake of NVs and Apo-NVs by BMSCs; NVs and Apo-NVs was labeled with DID (red). Phalloidin (green) was used to stain the cytoskeleton. Cell nuclei were stained with DAPI (blue). (D) Cell viability of BMSCs after 72 h treatment with NVs or Apo-NVs, measured by CCK-8 assay. (E) Apoptosis assay using Annexin V-FITC/PI staining. (F) Live/dead staining of BMSCs following treatment.

Initial characterization confirmed that the isolated BMSCs exhibited a characteristic spindle-shaped, fibroblastic morphology (Fig. 3A) and expressed standard surface marker profiles (CD11b^−^/CD45^-^, CD44^+^/CD29^+^), as validated by flow cytometric analysis (Fig. 3B). We first established the biocompatibility of the vesicles with BMSCs. Confocal microscopy and flow cytometric analysis confirmed the efficient cellular uptake of both NVs and Apo-NVs by BMSCs (Fig. 3C–Fig. S1), indicating their potential for intracellular signaling modulation. Critically, a suite of viability assays—including CCK-8 (Fig. 3D), Annexin V-FITC/PI apoptosis staining (Fig. 3E), and Calcein-AM/PI live/dead staining (Fig. 3F)—collectively demonstrated that co-culture with NVs or Apo-NVs for 72 h resulted in no significant cytotoxicity.

**Fig. 4:**
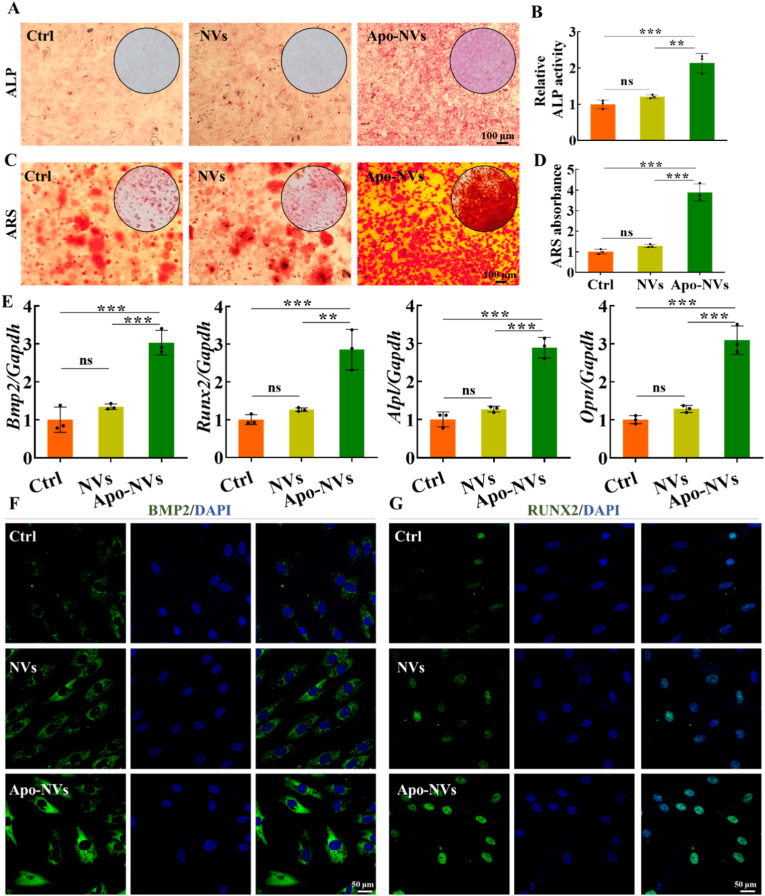
Pro-osteogenic effects of Apo-NVs on BMSCs. (A, B) ALP staining and ALP activity assays after 7 days of osteogenic induction. (C, D) Alizarin Red S staining and quantitative analysis of mineralized nodules after 14 days of osteogenic induction. (E) qPCR analysis of osteogenic gene expression (*Bmp2, Runx2, Alpl, Opn*). (F, G) Immunofluorescence staining of BMP2 and RUNX2 in BMSCs. Data represent mean ± SD; ∗∗∗p < 0.001, ∗∗p < 0.01; ns, not significant.

Having confirmed their biosafety, we next investigated the functional impact of Apo-NVs on the osteogenic differentiation cascade. Early osteogenic commitment, assessed by ALP activity after 7 days of induction, was markedly enhanced in BMSCs treated with Apo-NVs compared to both control and NVs-treated groups, as evidenced by more intense enzymatic staining (Fig. 4A and B). Furthermore, late-stage osteogenic maturation, evaluated by extracellular matrix mineralization via ARS staining at day 14, revealed significantly greater calcium nodule deposition in the Apo-NVs group (Fig. 4C and D). This robust promotion of osteogenesis was further substantiated at the molecular level. qPCR analysis showed a significant upregulation of key osteogenic transcriptions factors and marker genes, including *Bmp2, Runx2, Opn* and *Alpl,* in BMSCs exposed to Apo-NVs (Fig. 4E). Consistent with the gene expression data, immunofluorescence staining visually confirmed a pronounced increase in the protein expression of the critical osteogenic regulators BMP2 and RUNX2 (Fig. 4F, G and Fig. S2).

These data compellingly demonstrate that Apo-NVs possess an intrinsic osteoinductive capacity. They not only are biocompatible and efficiently internalized by BMSCs but also actively orchestrate the osteogenic differentiation program, accelerating both its early and terminal phases. This direct enhancement of BMSC function, coupled with their previously established immunomodulatory properties, positions Apo-NVs as a multifaceted therapeutic agent capable of synchronously targeting the immune and osteogenic components of the bone regeneration.

### 3.4 Angiogenic effect of Apo-NVs in vitro

The establishment of a robust and functional vascular network is a cornerstone of successful bone regeneration, ensuring the delivery of oxygen, nutrients, and osteoprogenitor cells to the defect site. Given the documented role of apoptotic cell-derived components in promoting angiogenesis—attributed to surface-exposed phosphatidylserine and other pro-angiogenic signaling molecules [13,36]—we hypothesized that our biomimetic Apo-NVs could inherit these properties and serve as potent inducers of new blood vessel formation.

**Fig. 5:**
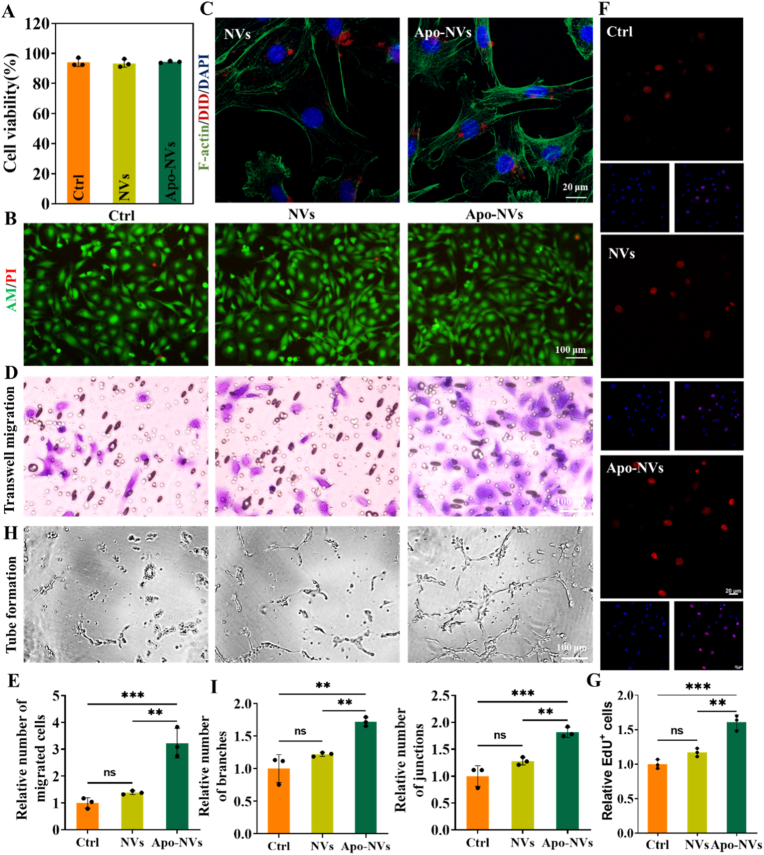
Pro-angiogenic effects of Apo-NVs on HUVECs. (A) CCK8 analysis of NVs and Apo-NVs treated HUVECs. (B) Live/dead staining of HUVECs after treatment with NVs or Apo-NVs. (C) Cellular uptake of NVs and Apo-NVs by HUVECs; NVs and Apo-NVs was labeled with DID (red). Phalloidin (green) was used to stain the cytoskeleton. Cell nuclei were stained with DAPI (blue). (D) Transwell migration assay. (E) Quantitative analysis of migrated cells. (F) EdU proliferation assay. (G) Relative proportion of EdU-positive cells in Fig. 5F. (H) Tube formation assay on Matrigel. (I) Quantitative analysis of tubular network parameters (junctions, branches). Data are presented as mean ± SD; ∗∗∗p < 0.001, ∗∗p < 0.01; ns, not significant.

CCK-8 assay (Fig. 5A) and Live/dead staining (Fig. 5B) and unequivocally demonstrated that co-culture with NVs or Apo-NVs did not compromise the viability or metabolic activity of HUVECs. Furthermore, confocal microscopy and flow cytometric analysis confirmed the efficient internalization of both vesicle types by HUVECs (Fig. 5C–Fig. S1), suggesting a potential route for direct intracellular signaling.

To investigate the angiogenesis modulated by Apo-NVs, we performed a series of functional assays. The initial migratory capacity of endothelial cells, a critical step in vessel sprouting, was significantly augmented by Apo-NVs treatment, as evidenced by a Transwell migration assay which showed a markedly increased number of migrated HUVECs compared to control and NVs-treated groups (Fig. 5D and E). Subsequently, a Click-iT EdU proliferation assay revealed that Apo-NVs potently stimulated HUVEC proliferation (Fig. 5F and G), indicating their capacity to expand the endothelial cell population, a prerequisite for the subsequent stages of vessel assembly. The most compelling evidence came from a Matrigel-based tube formation assay, which models the final stage of endothelial network assembly. HUVECs pre-treated with Apo-NVs generated an extensive, intricately connected tubular network, characterized by a significant increase in the number of junctions and branches compared to the sparse and disrupted structures observed in the control and NVs groups (Fig. 5H and I).

Collectively, these data delineate a comprehensive pro-angiogenic role for Apo-NVs. They are not only biocompatible and efficiently internalized by endothelial cells but also functionally potentiate the entire angiogenic cascade—enhancing cell migration, proliferation, and ultimately, the self-organization into capillary-like structures. This robust stimulation of in vitro vasculogenesis, coupled with their established immunomodulatory and osteoinductive properties, firmly establishes Apo-NVs as a multifaceted regenerative nanoplatform capable of synchronously targeting the critical triumvirate of angiogenesis, osteogenesis, and inflammation resolution for advanced bone tissue engineering.

### 3.5 In vivo bone regeneration promoted by GelMA–Apo-NVs

**Fig. 6:**
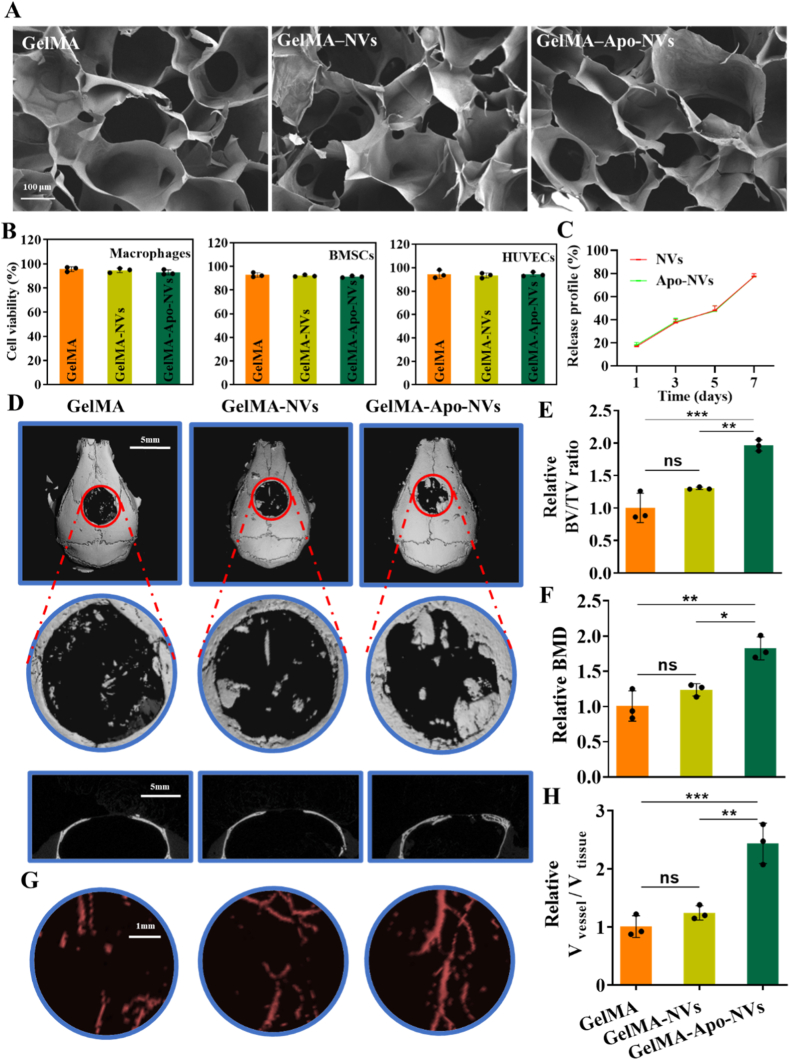
Micro-CT evaluation of calvarial bone repair. (A) SEM images of GelMA, GelMA–NVs and GelMA–Apo-NVs. (B) CCK8 analysis of GelMA, GelMA–NVs and GelMA–Apo-NVs treated macrophages, BMSCs and HUVECs; (C) Release profile of NVs and Apo-NVs loaded by GelMA. (D) Representative 3D-reconstructed micro-CT images of calvarial defects. (E, F) Quantitative analysis of bone volume/tissue volume (BV/TV) and bone mineral density (BMD). (G) Micro-CT angiography showing neovascularization within defect sites. (H) Quantification of vascular density. Data represent mean ± SD; ∗∗∗p < 0.001, ∗∗p < 0.01, ∗p < 0.05; ns, not significant.

Having established the multifaceted bioactivities of Apo-NVs in vitro, we next sought to evaluate their therapeutic efficacy in vivo. To achieve localized and sustained delivery, Apo-NVs were incorporated into GelMA hydrogel—a widely adopted biomaterial in bone tissue engineering celebrated for its biocompatibility, tunable physico-mechanical properties, and ability to mimic the native bone extracellular matrix [37,38]—forming a bioactive composite scaffold (GelMA–Apo-NVs). SEM analysis of the resulting GelMA–Apo-NVs hydrogel revealed a highly interconnected and uniformly porous microstructure, which is considered conducive to cellular infiltration, nutrient exchange, and vascular ingrowth (Fig. 6A). Prior to in vivo implantation, the cytocompatibility of the composite hydrogel was confirmed via CCK-8 assay, with both GelMA-NVs and GelMA–Apo-NVs groups showing no adverse effects on cell viability (Fig. 6B). Furthermore, the release profile of vesicles from the GelMA matrix was evaluated, demonstrating a comparable and sustained release kinetic for both NVs and Apo-NVs, which is crucial for long-term therapeutic action (Fig. 6C).

The regenerative potential of this composite scaffold was rigorously assessed in a murine model of critical-sized calvarial defect. After a 12-week implantation period, micro-computed tomography was employed for three-dimensional evaluation of bone repair. Representative 3D-reconstructed images (Fig. 6A) revealed minimal bone ingrowth in the empty GelMA and GelMA-NVs groups. In contrast, defects treated with the GelMA–Apo-NVs hydrogel exhibited significant new bone formation. Quantitative morphometric analysis corroborated these observations, showing a statistically significant increase in both BV/TV and BMD in the GelMA–Apo-NVs group compared to all other groups (Fig. 6B and C), underscoring its superior osteogenic efficacy.

Given the intimate coupling of angiogenesis and osteogenesis, we investigated the neovascularization within the defect site using micro-CT angiography following Microfil perfusion. The results revealed a remarkably denser and more highly interconnected vascular network penetrating the defect area in the GelMA–Apo-NVs group (Fig. 6D and E). This enhanced vascularization provides a plausible explanation for the robust bone regeneration observed, as it ensures adequate nutrient and osteoprogenitor supply.

**Fig. 7:**
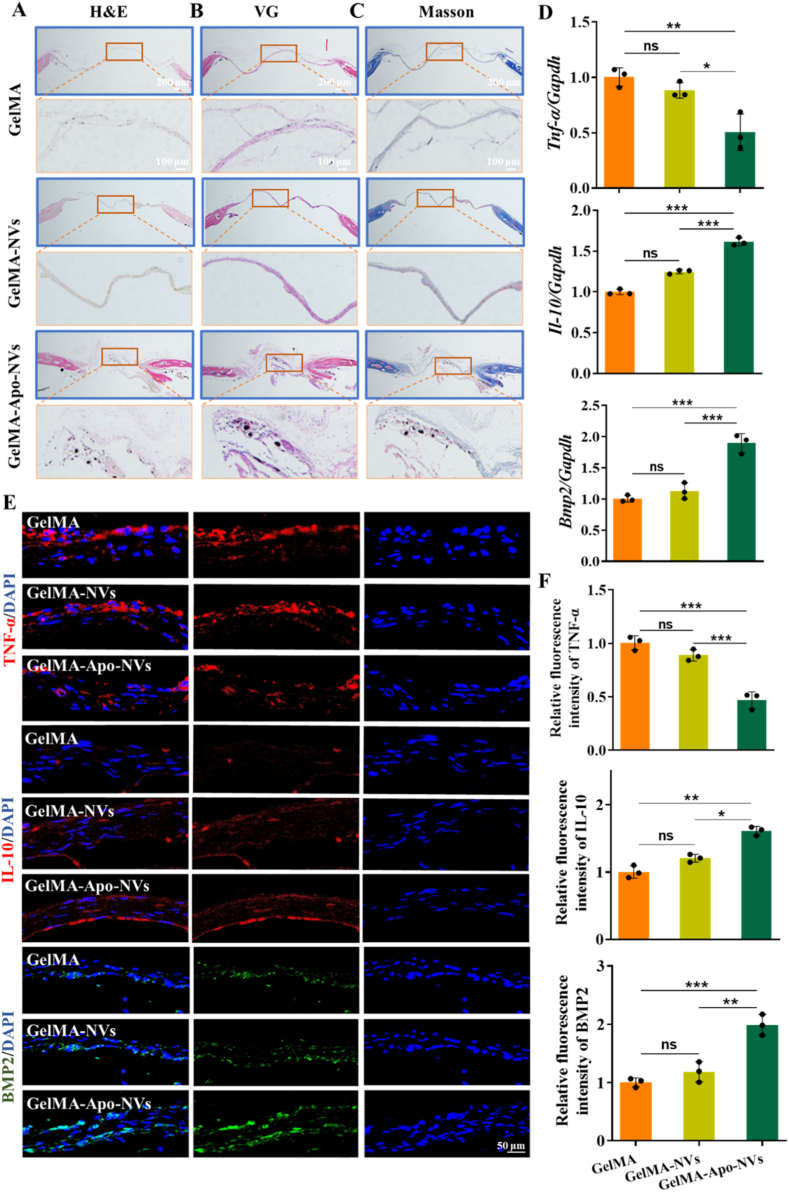
Histological and immunofluorescence assessment of bone regeneration. (A–C) H&E, Masson's trichrome, and Van Gieson staining of calvarial sections. New bone and collagen are stained red and blue, respectively. (D) qPCR analysis of *Tnf-α* and *Bmp2* expression in defect areas. (E) Immunofluorescence staining of TNF-α, IL-10 and BMP2. (F) Quantitative fluorescence intensity analysis of TNF-α, IL-10 and BMP2. Data represent mean ± SD; ∗∗∗p < 0.001, ∗∗p < 0.01, ∗p < 0.05; ns, not significant.

Histological analysis provided further evidence at the tissue level. Staining with H&E, Masson's trichrome, and VG (Fig. 7A–C) delineated the extent of new bone and collagen matrix deposition. The GelMA–Apo-NVs group displayed the most extensive and mature osteoid tissue, stained in red with VG, alongside well-organized collagen fibers (blue in Masson's trichrome). Finally, to gain insight into the local molecular microenvironment, we performed qPCR and immunofluorescence analysis on harvested defect tissues. The results indicated a significant downregulation of the pro-inflammatory cytokine TNF-α and a concurrent upregulation of anti-inflammatory cytokine IL-10 and osteogenic factor BMP2 in the GelMA–Apo-NVs group (Fig. 7D–F). To further validate angiogenesis at the tissue level, we performed immunofluorescence staining of vascular markers CD31 in defect sections. Fig. S3 demonstrated significantly increased expression level of CD31 in the GelMA–Apo-NVs group compared with GelMA and GelMA-NVs groups. These results confirm that the hydrogel composite not only builds structural bone but also actively fosters a pro-regenerative microenvironment by modulating local immune and osteogenic responses.

### 3.6 Mechanistic insights into bone regeneration via transcriptomic profiling

**Fig. 8:**
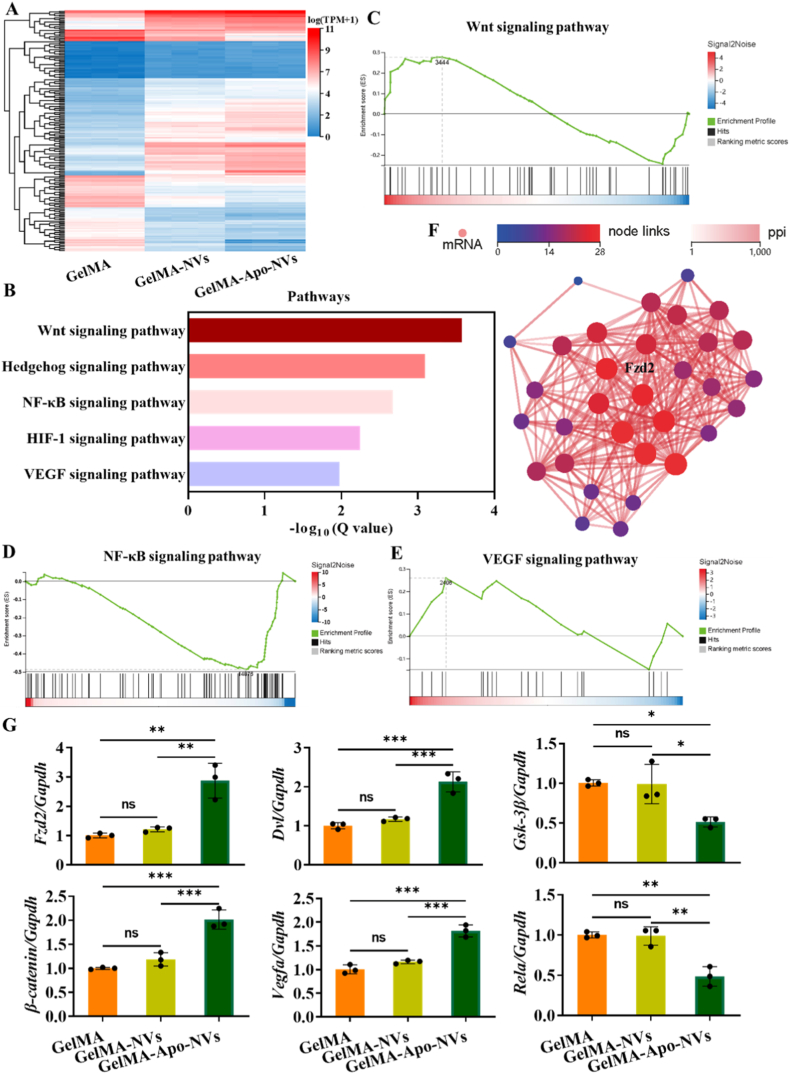
Transcriptomic analysis of regenerative mechanisms. (A) Heatmap of differentially expressed genes (DEGs) between groups. (B) KEGG pathway enrichment analysis of DEGs. (C-E) Gene Set Enrichment Analysis (GSEA) of the Wnt, NF-κB, VEGF signaling pathway. (F) Protein–protein interaction (PPI) network highlighting key regulators. (G) qPCR validation of key genes involved in regenerative signaling. Data represent mean ± SD; ∗∗∗p < 0.001, ∗∗p < 0.01, ∗p < 0.05; ns, not significant.

To elucidate the molecular mechanisms underlying the enhanced bone regeneration mediated by Apo-NVs, transcriptomic profiling via RNA sequencing was conducted. Fig. 8A reveals distinct clustering and transcriptional divergence among the experimental groups, as illustrated by the heatmap analysis of differentially expressed genes (DEGs). Notably, the GelMA–Apo-NVs group exhibits a unique gene expression profile clearly separated from GelMA group, underscoring the substantial modulation of global transcription induced by Apo-NVs. KEGG enrichment analysis of 188 differentially expressed genes highlighted the involvement of multiple signaling pathways, including Wnt, Hedgehog, NF-κB, HIF-1, and VEGF (Fig. 8B).

Particularly noteworthy is the central role of the Wnt/β-catenin pathway, which serves as a master regulator of osteogenic differentiation. Upon binding of Wnt ligands (e.g., Wnt3a, Wnt10b) to Frizzled receptors and LRP5/6 co-receptors, the degradation complex of β-catenin is inhibited, leading to its cytoplasmic stabilization and subsequent nuclear translocation. In the nucleus, β-catenin associates with TCF/LEF transcription factors to activate key osteogenic genes, including *Runx2*—the earliest and most critical transcription factor governing osteoblast commitment—and Osterix (*Osx*), which is essential for osteoblast maturation [39,40]. This cascade ultimately promotes the differentiation of mesenchymal stem cells into osteoblasts, enhances osteoblastic activity and function, and suppresses apoptosis.

Simultaneously, the Wnt/β-catenin pathway exerts vital functions in endothelial cells (ECs). Nuclear translocation of β-catenin also occurs in ECs, where the β-catenin/TCF complex drives the expression of pro-angiogenic genes. Mechanistically, nuclear β-catenin directly binds to the promoter region of the *Vegfa* gene, thereby initiating its transcription and upregulating mRNA expression levels [[41], [42], [43]]. This establishes the Wnt/β-catenin pathway as a critical upstream regulator of VEGF production. Consequently, the β-catenin/TCF complex orchestrates the expression of key angiogenic factors including VEGF, its receptor VEGFR2, Angiopoietin-2 (Ang-2), and matrix metalloproteinases (MMPs) [[44], [45], [46]]. These facilitate endothelial proliferation, migration, tube formation, and extracellular matrix remodeling, collectively promoting angiogenesis. Furthermore, Wnt/β-catenin signaling contributes to immunomodulation by promoting M2 macrophage polarization, thereby suppressing excessive inflammation and fostering a regenerative microenvironment. This effect is partly mediated through molecular interference with the NF-κB pathway—a key mechanism involving the direct binding of β-catenin to critical NF-κB components such as p65 (*Rela*). This interaction prevents nuclear translocation of p65 or suppresses its transcriptional activity [47,48], thereby reducing the production of pro-inflammatory cytokines (e.g., TNF-α, IL-1β, IL-6) and enhancing the expression of anti-inflammatory mediators such as IL-10.

GSEA further affirmed significant alterations in Wnt, NF-κB, and VEGF signaling pathways (Fig. 8C–E), supporting their coordinated role in coupling immune regulation, osteogenesis, and angiogenesis. PPI network analysis identified *Fzd2*, a crucial Wnt receptor, as a hub gene within this pathway (Fig. 8F). The binding of Wnt ligands (e.g., WNT3A) to FZD2 stabilizes the receptor complex, leading to the subsequent activation of the Wnt/β-catenin cascade. This signaling axis is instrumental in driving the proliferation and differentiation of osteoblasts. Complementary mechanistic investigation (Fig. 8G) demonstrated that Apo-NVs activated *Fzd2*, initiating a downstream signaling cascade that involves *Dvl* and *Gsk-3β*, resulting in the stabilization and nuclear translocation of *β-catenin*. This process ultimately modulated the expression of pivotal effector genes including *Vegfa* (associated with angiogenesis) and *Rela* (a key subunit of NF-κB), confirming that Apo-NVs activated a multi-pathway regenerative signaling centered around Wnt/β-catenin pathway, which orchestrated the interplay among inflammatory response, bone formation, and vascularization during cranial defect repair.

Apo-NVs in this study were engineered from plasma membranes of early apoptotic T lymphocytes and therefore are expected to primarily convey membrane-associated apoptotic cues. Among these, phosphatidylserine externalization is a hallmark feature that promotes preferential uptake by phagocytes and initiates efferocytosis-associated pro-resolving programs, including increased IL-10/TGF-β and reduced pro-inflammatory cytokines [33,49]. In macrophages, efferocytosis signaling through receptors such as MerTK has been shown to block NF-κB signaling, thereby suppressing inflammatory cytokine production and favoring inflammation resolution [50,51]. This provides a mechanistic basis for our observed downregulation of inflammatory readouts (e.g., TNF-α) and the transcriptomic enrichment indicative of NF-κB attenuation.

Notably, the immuno-osteogenic coupling inferred from the RNA-seq results is consistent with established crosstalk between NF-κB and β-catenin pathways. NF-κB can restrain osteogenic differentiation in inflammatory settings, in part by antagonizing β-catenin signaling, whereas relieving NF-κB pressure can benefit osteogenic programs [48,52]. In parallel with immune resolution, we observed activation of Wnt/β-catenin–associated signatures and upregulation of Vegfa, supporting an angiogenesis–osteogenesis coupling axis. Given that efferocytosis pathways can engage PI3K/Akt signaling modules and that PI3K/Akt activity is functionally connected to GSK-3β/β-catenin regulation, we propose that Apo-NVs–initiated pro-resolving signaling may converge with Wnt/β-catenin activation to orchestrate the immune–vascular–osteogenic triad observed in our model [48,53,54].

Beyond PS, apoptosis-associated immunomodulation may also involve surface protein determinants remodeled during apoptosis; thus, PS should be viewed as a major but not exclusive contributor [55]. Additionally, the T-cell membrane origin provides adhesion machinery such as LFA-1, which mediates leukocyte adhesion and transendothelial interactions and may enhance interface with inflamed endothelium/immune compartments, potentially favoring local retention and intercellular communication after implantation [56,57]. Previous studies also indicate that PS-dependent uptake pathways can operate in endothelial cells, supporting the plausibility of Apo-NVs contributions to angiogenic phenotypes observed in vitro and in vivo [58,59]. Collectively, these observations support that Apo-NVs membrane cues (notably PS exposure) promote efferocytosis-associated immune resolution, thereby facilitating Wnt/β-catenin–dependent osteogenic programs and Vegfa-linked vascular responses.

## 4 Conclusion

**Scheme 1:**
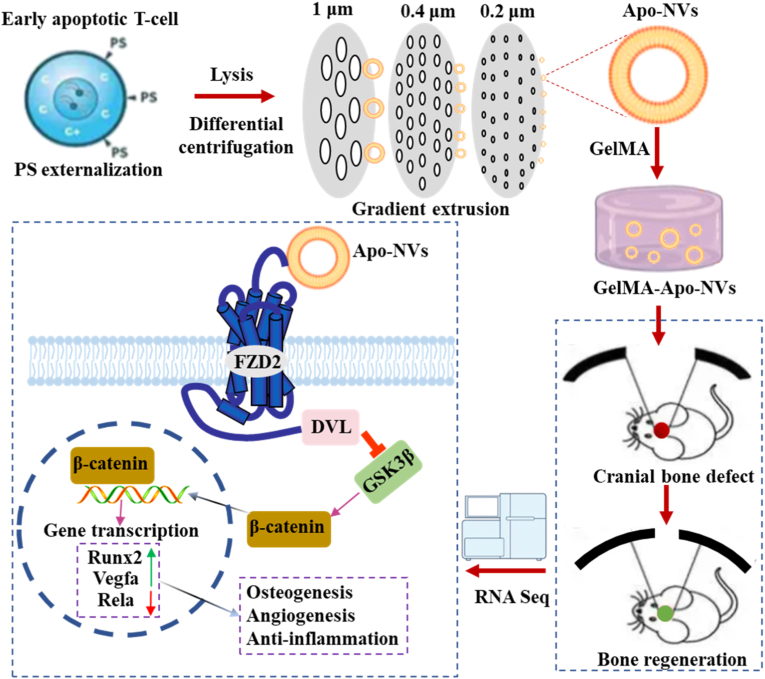
Schematic diagram of Apo-NVs promoting bone regeneration.

In this investigation, we established a protocol for generating Apo-NVs that retain crucial apoptotic membrane signatures, notably phosphatidylserine exposure. These Apo-NVs exhibit exceptional capabilities in modulating the local immune microenvironment by driving macrophage polarization towards an M2 reparative phenotype, directly enhancing the osteogenic differentiation of BMSCs, and potently stimulating angiogenesis in vitro. The incorporation of Apo-NVs into a GelMA hydrogel facilitated sustained release and significantly enhanced bone repair and vascularization in a critical-sized calvarial defect model, outperforming GelMA and GelMA-NVs groups. Crucially, RNA sequencing elucidated that Apo-NVs function as a regulator by activating the Wnt/β-catenin pathway. This signaling orchestrates a coupled regeneration process by simultaneously suooressing inflammation through NF-κB inhibition, directly promoting osteogenic gene expression, and transcriptionally inducing *Vegfa*-driven angiogenesis (Scheme 1). The Apo-NVs-based hydrogel, characterized by its bioactivity, safety, and mechanistic clarity, represents a highly promising strategy for the healing of challenging bone defects.

## CRediT authorship contribution statement

**Hao Pan:** Conceptualization, Data curation, Formal analysis, Funding acquisition, Investigation, Methodology, Project administration, Resources, Software, Supervision, Validation, Visualization, Writing – original draft, Writing – review & editing. **Min Zhang:** Conceptualization, Data curation, Formal analysis, Funding acquisition, Investigation, Methodology, Project administration, Resources, Software, Supervision, Validation, Visualization, Writing – original draft, Writing – review & editing. **Likai Chen:** Methodology, Resources, Supervision, Validation. **Haoze Zhu:** Methodology, Project administration, Supervision. **Siman Huang:** Supervision, Validation, Visualization. **Yueyue Huang:** Project administration, Resources, Software. **Yiyu Li:** Data curation, Formal analysis, Funding acquisition. **Zuchang Liu:** Methodology, Project administration. **Xiaokun Li:** Conceptualization, Data curation, Formal analysis, Funding acquisition, Investigation, Methodology, Project administration, Resources, Software, Supervision, Validation, Visualization, Writing – original draft, Writing – review & editing. **Cailong Liu:** Conceptualization, Data curation, Formal analysis, Funding acquisition, Investigation, Methodology, Project administration, Resources, Software, Supervision, Validation, Visualization, Writing – original draft, Writing – review & editing.

## Declaration of competing interest

The authors declare that they have no known competing financial interests or personal relationships that could have appeared to influence the work reported in this paper.

## Data Availability

Data will be made available on request.
