# Supplementary material for: Apoptotic-mimetic nanovesicles orchestrate immune–vascular–osteogenic crosstalk for critical-sized craniofacial bone regeneration
Source: Mater Today Bio. 2026 Apr 13;38:103119. doi: 10.1016/j.mtbio.2026.103119 (PMC13101605; doi:10.1016/j.mtbio.2026.103119)
Supplement: Multimedia component 1 [file mmc1.docx]

**Supplementary Material**

# Apoptotic-Mimetic Nanovesicles Orchestrate Immune–Vascular–Osteogenic Crosstalk for Critical-Sized Craniofacial Bone Regeneration

Hao Pan^1,2,#^, Min Zhang^5,#^, Likai Chen^1^, Haoze Zhu^1^, Siman Huang^1^, Yueyue Huang^3^, Yiyu Li^4^, Zuchang Liu^4^, Xiaokun Li^1,2^*, Cailong Liu^1^*

^1^ Department of Orthopaedic Surgery, Department of Wound Healing, The First Affiliated Hospital of Wenzhou Medical University, Wenzhou, Zhejiang 325000, China.

^2^ National Key Laboratory of Macromolecular Drugs and Large-scale Preparation, School of Pharmaceutical Sciences, Wenzhou Medical University, Wenzhou, Zhejiang, 325035, China.

^3^ Key Laboratory of Intelligent Treatment and Life Support for Critical Diseases of Zhejiang Province, Department of Intensive Care Unit, The First Affiliated Hospital of Wenzhou Medical University, Wenzhou, Zhejiang 325000, China.

^4^ Department of Clinical Biochemistry and Molecular Diagnostics, College of Medical Technology, Tianjin Medical University, Tianjin, 300203, China.

^5^ Shanghai Engineering Research Center of Tooth Restoration and Regeneration & Tongji Research Institute of Stomatology & Department of Dental Implantation, Shanghai Tongji Stomatological Hospital and Dental School, Tongji University, Shanghai, 200072, China.

^#^ These authors contributed equally to this work.

* Corresponding authors.

*E-mail addresses*: [profxiaokunli@163.com](mailto:profxiaokunli@163.com) (Xiaokun Li), [15888297757@163.com](mailto:15888297757@163.com) (Cailong Liu).


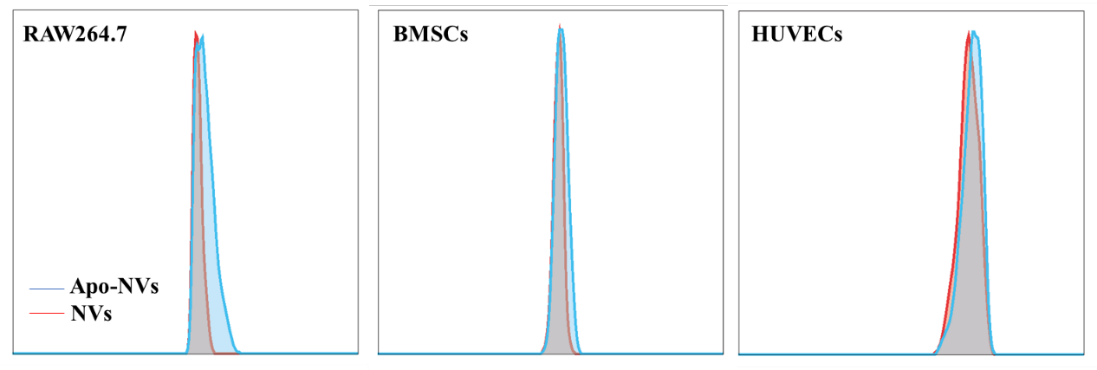


**Fig. S1.** Flow cytometry analysis of macrophages, BMSC and HUVECs phagocytosed with nanovesicles. The red filled curves mean NVs control and the blue filled curves represent the Apo-NVs.

**Fig. S2.** The relative fluorescence intensity of BMP2 and RUNX2 in Figure 4F and 4G (n=3, ***p < 0.001, **p < 0.01; ns, not significant).


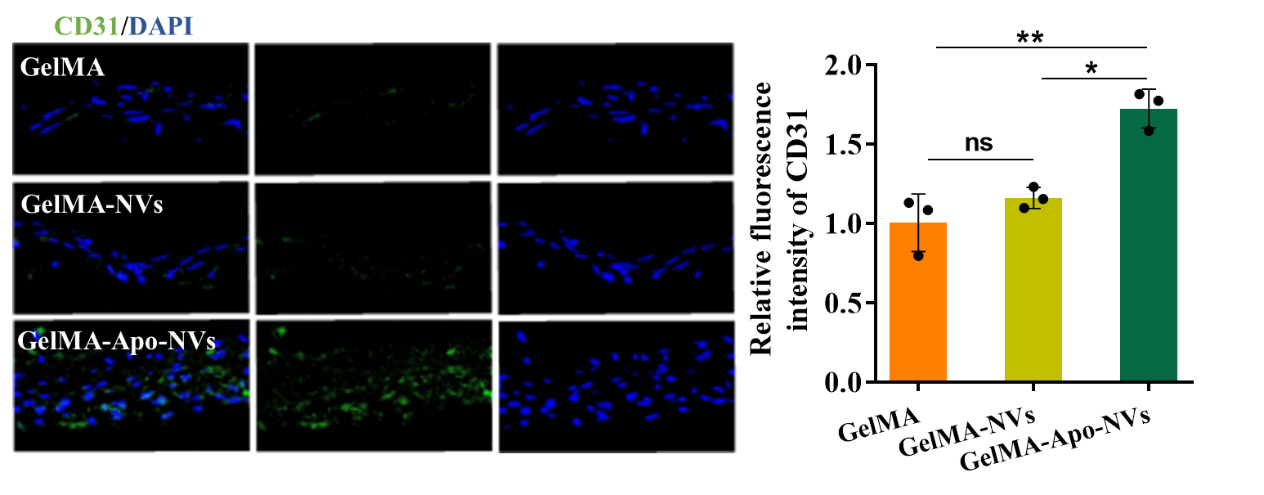


**Fig. S3.** Immunofluorescence staining and quantitative analysis of CD31. (n=3, ***p < 0.001, **p < 0.01; ns, not significant).

Table S1. qPCR primer of Macrophages (RAW264.7).

| Gene | Forward primer | Reverse primer |
| --- | --- | --- |
| *Gapdh* | CATCACTGCCACCCAGAAGACTG | ATGCCAGTGAGCTTCCCGTTCAG |
| *Tnf-α* | ATGCTGGGACAGTGACCTGG | CCTTGATGGTGGTGCATGAG |
| *Il-1β* | TGGACCTTCCAGGATGAGGACA | GTTCATCTCGGAGCCTGTAGTG |
| *Tgf-β* | TGATACGCCTGAGTGGCTGTCT | CACAAGAGCAGTGAGCGCTGAA |
| *Il-10* | GCTGGACAACATACTGCTAACC | ATTTCCGATAAGGCTTGGCAA |

Table S2. qPCR primer of BMSCs.

| Gene | Forward primer | Reverse primer |
| --- | --- | --- |
| *Gapdh* | CATCACTGCCACCCAGAAGACTG | ATGCCAGTGAGCTTCCCGTTCAG |
| *Bmp2* | AACACCGTGCGCAGCTTCCATC | CGGAAGATCTGGAGTTCTGCAG |
| *Alpl* | CCAGAAAGACACCTTGACTGTGG | TCTTGTCCGTGTCGCTCACCAT |
| *Opn* | GCTTGGCTTATGGACTGAGGTC | CCTTAGACTCACCGCTCTTCATG |
| *Runx2* | CCTGAACTCTGCACCAAGTCCT | TCATCTGGCTCAGATAGGAGGG |

Table S3. qPCR primer of tissue.

| Gene | Forward primer | Reverse primer |
| --- | --- | --- |
| *Gapdh* | CATCACTGCCACCCAGAAGACTG | ATGCCAGTGAGCTTCCCGTTCAG |
| *Tnf-α* | ATGCTGGGACAGTGACCTGG | CCTTGATGGTGGTGCATGAG |
| *Il-10* | GCTGGACAACATACTGCTAACC | ATTTCCGATAAGGCTTGGCAA |
| *Bmp2* | AACACCGTGCGCAGCTTCCATC | CGGAAGATCTGGAGTTCTGCAG |
| *Dvl* | ATCACACGCACCAGCTCTTCCT | AGTCCTGAGTCTGGCAACTGCA |
| *Gsk-3β* | GAGCCACTGATTACACGTCCAG | CCAACTGATCCACACCACTGTC |
| *β-catenin* | GTTCGCCTTCATTATGGACTGCC | ATAGCACCCTGTTCCCGCAAAG |
| *Vegfa* | CTGCTGTAACGATGAAGCCCTG | GCTGTAGGAAGCTCATCTCTCC |
| *Rela* | TCCTGTTCGAGTCTCCATGCAG | GGTCTCATAGGTCCTTTTGCGC |
| *Fzd2* | CGCTTCCACTTTCTTCACGGTC | GGAGAACGAAGCCCGCAATGTA |
